# 752. Decreased Echinocandin Resistance among Candida glabrata over an Eleven-Year Period at a University Hospital

**DOI:** 10.1093/ofid/ofac492.037

**Published:** 2022-12-15

**Authors:** Nathan Raabe, Yanan Zhao, Padmaja Paderu, Ryan K Shields, Cornelius J Clancy, Minh-Hong Nguyen

**Affiliations:** University of Pittsburgh, Pittsburgh, Pennsylvania; 2Hackensack Meridian School of Medicine & Hackensack Meridian Health Center for Discovery and Innovation, Nutley, New Jersey; Center for Discovery and Innovation, Hackensack Meridian Health, Nutley, New Jersey; University of Pittsburgh, Pittsburgh, Pennsylvania; University of Pittsburgh, Pittsburgh, Pennsylvania; University of Pittsburgh, Pittsburgh, Pennsylvania

## Abstract

**Background:**

Invasive candidiasis (IC) is associated with significant morbidity and mortality. Echinocandins (ECH; caspofungin (CAS), micafungin (MFG), anidulafungin (AFG)) are first line therapy for IC. ECH resistance has emerged widely, most commonly among Candida glabrata. At some centers, > 10% of C. glabrata are ECH resistant. We reported ECH resistance in 8% of C. glabrata at our hospital from 2008–2014, which prompted stewardship strategies to limit prolonged ECH treatment courses. We evaluated trends of ECH resistance from Jan 2010-Dec 2020 among C. glabrata clinical isolates, and assayed isolates for FKS hot spot (HS) mutations.

**Methods:**

We mined electronic medical records for ECH MIC data against *C. glabrata* clinical isolates at UPMC. We defined ECH resistance using Clinical and Laboratory Standards Institute clinical breakpoints. Isolates resistant to any ECH underwent *FKS* Sanger sequencing. Statistical analysis was performed using Stata.

**Results:**

Overall CAS, AFG and MFG resistance rates among 516 isolates were 4%, 3%, and 2.5%, respectively. There was no significant difference in resistance rates between blood and non-blood sites (Table 1). There was a significant decrease in MFG resistance over the 12-year period (Fig 1). Time series regression analysis showed a decrease in rate of MFG resistance of 0.73% per year (p=.007). Trends for decreases in AFG and CAS resistance rates were also noted (p=.1 and p=.17). Similar analyses showed annual decreases in MFG MICs (0.019 μg/mL per year; p=.07). The average percentage of AFG resistant isolates was significantly lower in 2017–2020 than in 2012–2016 (p=.04); similar trends were evident for MFG (p=.07) and CAS (p=.08) (Fig 2). Sanger sequencing of FKS1/2 was performed for 27 isolates resistant to ≥1 ECH. Mutations were found in 44% (12/27) of isolates: 5 mutations in HS1 *FKS1*, 1 in HS2 *FKS1*, 5 in HS1 *FKS2* and 1 in HS2 *FKS2*. Using both CAS and AFG MICs was most predictive of FKS mutations (Fig. 3).

**Conclusion:**

ECH resistance among *C. glabrata* stabilized or decreased over 11-years at our hospital, in keeping with stewardship interventions to reduce prolonged use of ECHs. These results demonstrate that it is feasible to conserve ECH susceptibility.

**Disclosures:**

**Cornelius J. Clancy, MD**, receives research funding paid to his institution from Astellas and Merck: Grant/Research Support|serves as an advisory Board member for Astellas, Cidara, and Scynexis, served on the advisory board for Merck, Qpex Biopharma, and Shionogi: Advisor/Consultant|Venatorx and Needham & Associates: Advisor/Consultant **Minh-Hong Nguyen, MD**, QPX: Grant/Research Support.

